# Influence of T Cell-Mediated Immune Surveillance on Somatic Mutation Occurrences in Melanoma

**DOI:** 10.3389/fimmu.2021.703821

**Published:** 2022-01-17

**Authors:** Chongming Jiang, Evelien Schaafsma, Wei Hong, Yanding Zhao, Ken Zhu, Cheng-Chi Chao, Chao Cheng

**Affiliations:** ^1^ Department of Medicine, Baylor College of Medicine, Houston, TX, United States; ^2^ Department of Molecular and Systems Biology, Dartmouth College, Hanover, NH, United States; ^3^ Medical School, UT Southwestern Medical Center, Dallas, TX, United States; ^4^ Antibody Discovery, Chempartner Corporation, South San Francisco, CA, United States; ^5^ Dan L. Duncan Comprehensive Cancer Center, Baylor College of Medicine, Houston, TX, United States; ^6^ Department of Biomedical Data Science, Geisel School of Medicine at Dartmouth, Lebanon, NH, United States; ^7^ The Institute for Clinical and Translational Research, Baylor College of Medicine, Houston, TX, United States

**Keywords:** Melanoma, recurrent mutation, immunosurveillance, neoantigen, antigen presentation

## Abstract

**Background:**

Neoantigens are presented on the cancer cell surface by peptide-restricted human leukocyte antigen (HLA) proteins and can subsequently activate cognate T cells. It has been hypothesized that the observed somatic mutations in tumors are shaped by immunosurveillance.

**Methods:**

We investigated all somatic mutations identified in The Cancer Genome Atlas (TCGA) Skin Cutaneous Melanoma (SKCM) samples. By applying a computational algorithm, we calculated the binding affinity of the resulting neo-peptides and their corresponding wild-type peptides with the major histocompatibility complex (MHC) Class I complex. We then examined the relationship between binding affinity alterations and mutation frequency.

**Results:**

Our results show that neoantigens derived from recurrent mutations tend to have lower binding affinities with the MHC Class I complex compared to peptides from non-recurrent mutations. Tumor samples harboring recurrent SKCM mutations exhibited lower immune infiltration levels, indicating a relatively colder immune microenvironment.

**Conclusions:**

These results suggested that the occurrences of somatic mutations in melanoma have been shaped by immunosurveillance. Mutations that lead to neoantigens with high MHC class I binding affinity are more likely to be eliminated and thus are less likely to be present in tumors.

## Introduction

Cancer is a genetic disease caused by genomic abnormalities including somatic mutations, which result in mutated antigens (i.e., neoantigens). Neoantigens derived from non-synonymous mutations can be recognized, bound, and presented on the tumor cell surface by major histocompatibility complex (MHC) proteins. T cells can recognize and attack tumor cells presenting these neoantigens, which is known as T cell-mediated cancer immunosurveillance (1, 2). However, tumors can develop different strategies to avoid recognition and elimination by the immune system (3–5).

Evolving neoplasms accumulate non-synonymous mutations at a high rate, leading to the expression of antigenic epitopes that might be recognized by the immune system (6). According to the theory of immunosurveillance, a functional immune system can recognize and eliminate tumor cells harboring antigenic mutations (1, 7). Tumor cells presenting immune-activating neoantigens are more likely to be eliminated through T-cell recognition as compared to non-immunogenic mutations (8–10). Consequently, somatic mutations abrogating essential immune functions (e.g., mutations in B2M and HLA genes) are generally positively selected for in tumors and are common in different types of cancers (11). In contrast, the majority of somatic mutations are under negative selection during the tumorigenesis by immunosurveillance. A number of previous studies have shown that the immune system can exert strong selection pressure on neoantigens in both untreated and treated tumors (12–15). During lung cancer evolution, the immune system also exhibits neoantigen-editing in which cells with immunogenic mutations are eliminated (14). Indeed, the observed counts of neoantigens were unexpectedly low in some tumor types (16, 17), suggesting the impact of negative selection posed by immunosurveillance. However, other studies have suggested that neoantigen selection by the immune system becomes negligible in untreated tumor samples when considering mutational signatures (18, 19).

The potential impact of immunosurveillance on gene mutation abundance is determined by multiple factors, namely, the category of mutated genes (cancer driver or passenger genes), mutation frequency (12, 20–22), the expression level of the mutated genes (23), and HLA functionality (proficient or deficient) (24). The capacity of antigens to induce a CD8^+^ T cell immune response (antigenicity) is mainly determined by their binding affinity with the MHC class I (MHC-I) complex. In this study, we investigated the non-synonymous somatic mutations identified in the TCGA Skin Cutaneous Melanoma (SKCM) samples. We applied a computational pipeline to calculate the binding affinity of the resulting neoantigens with the MHC-I complex. As a control, we calculated the MHC-I binding affinity of the wide-type peptide. We found that neoantigens of recurrent mutations have significantly lower MHC-I binding affinity than those from non-recurrent mutations. These results were observed for somatic mutations presenting in both melanoma-specific driver and passenger genes. In addition, we investigated the potential influence of HLA genotypes and HLA gene deficiency (mutation and loss events). Our results suggested that the somatic mutation landscape in melanoma is shaped by T cell-mediated immunosurveillance.

## Results

### Quantification of the Effect of Non-Synonymous Mutations on MHC-I Binding Affinity

**Figure 1:**
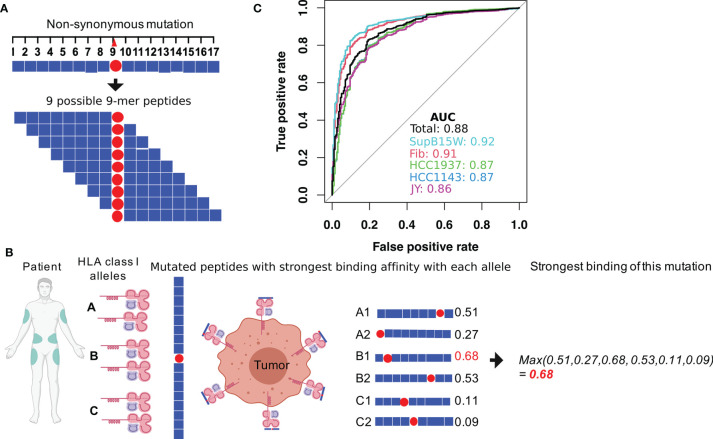
Calculation of the MHC-I binding affinity of the neoantigen derived from a non-synonymous mutation. **(A)** For each mutation, a total of 17 amino acids centering at the mutated site were considered. The binding affinities with MHC-I complex of the nine possible 9-mer peptides were calculated by using NetMHCPan4.0 (25). **(B)** During the MHC-I binding affinity calculation, patient-specific HLA genotypes were used. Each patient has two alleles for HLA-A, B, and C gene, resulting in six total alleles. For each allele, MHC-I binding affinities to the nine 9-mers were calculated. The final MHC-I binding affinity of the somatic mutation was calculated as the maximum value of the 9 (peptides) × 6 (alleles) binding scores. Figure created using BioRender (https://biorender.com/). **(C)** The performance of our MHC-I binding affinity pipeline was evaluated by classifying MHC binding and non-MHC binding epitopes in experimental data from previous studies (26, 27). The data provided peptide sequences for a collection of MHC binding and non-MHC binding epitopes in 5 different human cell lines (Fibroblast, SupB15W, JY, HCC1937, and HCC1143).

To investigate how T cell-mediated immunosurveillance shapes the mutational landscape in SKCM tumors, we calculated a residue-centric presentation score to quantify MHC-I binding affinity changes that resulted from non-synonymous mutations using the method described in **Figure 1**. First, for each non-synonymous mutation, we enumerated nine 9-mers (peptides with 9 amino acids) that overlap with the mutated residue (**Figure 1A**). We applied NetMHCPan4.0 (25) to these 9-mers to calculate their binding affinity with the MHC-I complex (**Figure 1B**) using six patient-specific HLA class I alleles (two HLA-A, two HLA-B, and two HLA-C genes). We decided on NetMHCPan4.0 for MHC-I binding affinity calculations due to its overall high and stable performance as demonstrated in previous studies (28–30). Each mutation-derived peptide received a prediction score within [0,1], with higher scores indicating higher binding affinities with the MHC-I complex. In total, 54 binding scores were calculated for the nine peptides that resulted from a mutation with the six HLA class I alleles. Second, we calculated the maximum value for each HLA allele to capture the peptide with the highest binding affinity, resulting in six HLA class I allele-specific affinity scores. Finally, we selected the maximum value of the six HLA class I allele-specific affinity scores to represent the best MHC-I complex presentation potential for peptides derived from a specific non-synonymous mutation (see *Materials and Methods* section). In **Figure 1C**, we evaluated the prediction accuracy of this pipeline by applying it to a combined benchmark data (**Supplementary Table 1**), containing experimentally identified MHC-I binding peptides (27) and non-binding control peptides (26). As shown, our pipeline can accurately classify these two types of peptides with an average AUC (area under receiver operating characteristic curve) score of 0.88 (**Figure 1C**). After verifying the robustness of the pipeline, we applied it to a total of 122,603 non-synonymous mutations identified from 345 TCGA SKCM tumor samples (**Supplementary Table 2**).

### Neoantigens Derived From Recurrent Mutations Have Lower Binding Affinities Than Those From Non-Recurrent Mutations

**Figure 2:**
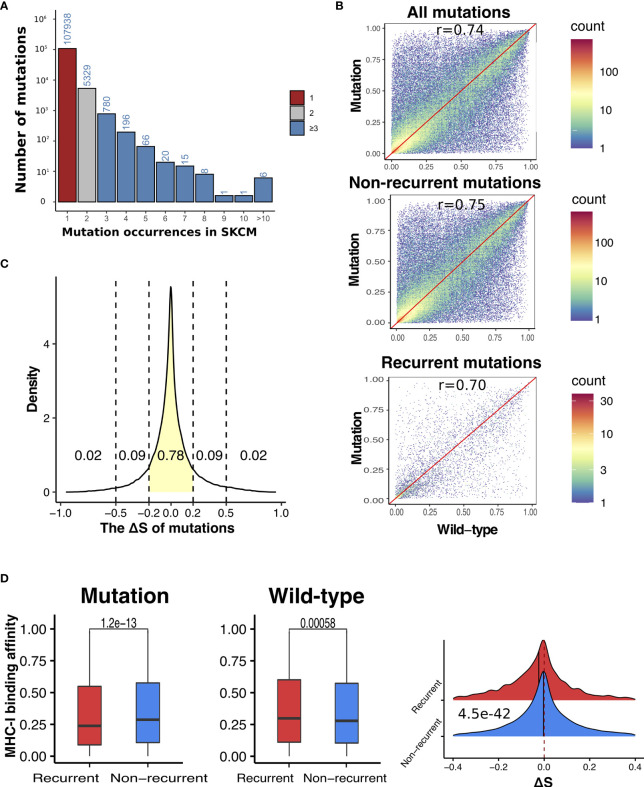
Recurrent somatic mutations have significantly lower ΔS than non-recurrent mutations in SKCM. **(A)** The distribution of occurrences of non-synonymous mutations in SKCM. Mutation count was calculated as the number of melanoma patients harboring a specific somatic mutation. Based on the distribution, we defined recurrent mutations (blue) as those presenting in at least three melanoma samples, and non-recurrent mutations (red) as those presenting in only one sample. **(B)** Correlations between antigens derived from mutations (all, non-recurrent, and recurrent) and the corresponding wide-type in MHC-I binding affinities. **(C)** The density plot of ΔS, which was defined as the difference between the MHC-I binding score of mutant and wild-type antigens. **(D)** Comparison between recurrent vs non-recurrent mutations in the MHC-I binding affinity with antigens derived from mutations (left) and the wild-type (middle), as well their difference (ΔS) (right). In all plots, P-values were calculated using two-sided Wilcoxon rank-sum test and adjusted for multiple testing by the Holm–Bonferroni method.

We hypothesize that somatic mutations that result in neoantigen with high MHC-I binding affinity are subject to negative selection *via* immunosurveillance and are therefore less likely to be present at high frequencies in tumor samples. To test this hypothesis, we divided non-synonymous (NS) mutations identified in the TCGA SKCM samples (29) into recurrent (occurred in at least three samples) and non-recurrent mutations (occurred in only one sample) (**Figure 2A**). In total, we identified 5,677 recurrent and 97,618 non-recurrent NS mutations. For each mutation, we calculated the aforementioned binding score that indicates the putative highest possible affinity score a mutation-derived peptide can receive given the HLA-A, B, and C allele type of a patient (see *Materials and Methods* for details). First, we calculated MHC-I binding affinity correlations between mutation-derived peptides and the corresponding wild-type peptides. Overall, we observed fairly high correlations with recurrent mutations showing slightly lower correlations than non-recurrent mutations (r = 0.70 versus 0.75) (**Figure 2B**), indicating larger differences in MHC class I binding affinities upon mutation in recurrent mutations as compared to non-recurrent mutations. To quantify the effect of mutations on binding affinity, we calculated binding score differences between mutant and wild-type peptides to obtain ΔS for each mutation. The ΔS takes a value within [−1, 1] with the distribution shown in **Figure 2C**.

As shown in **Figure 2D**, recurrent mutations showed significantly lower binding affinity with MHC-I complex in their mutation-derived peptides than non-recurrent mutations (adjust P-value (*P_adj_*) = 1.2e−13). We also compared the MHC-I binding scores of the corresponding wild-type peptides and found that the recurrent mutations showed significantly higher MHC-I binding scores than non-recurrent mutations (*P_adj_* = 0.0058). When ΔS values were compared, the recurrent mutations are significantly lower than non-recurrent mutations (*P_adj_* = 4.5e−42). Altogether, our results suggest that the presence of somatic mutations are likely shaped by immunosurveillance—tumor cells hosting mutations with higher MHC-I binding affinity are more likely to be eliminated and, as a consequence, these mutations are less to be present in tumors.

In addition to melanoma, we also applied the same analysis in 16 TCGA cancer types with available HLA genotypes and large sample size. Out of them, we found significantly ΔS reduction in recurrent (occurrence ≥3) than non-recurrent mutations (occurrence = 1) in 6 cancer types, breast cancer, cervical cancer, rectum adenocarcinoma, stomach adenocarcinoma, endometrial cancer, and kidney cancer (**Figure S1**). Some of the non-significant cancer types have relative low tumor mutation burden and therefore the numbers of recurrent mutations are small, which limited the statistical power for ΔS comparison.

### Somatic Mutations in Driver and Passenger Genes

**Figure 3:**
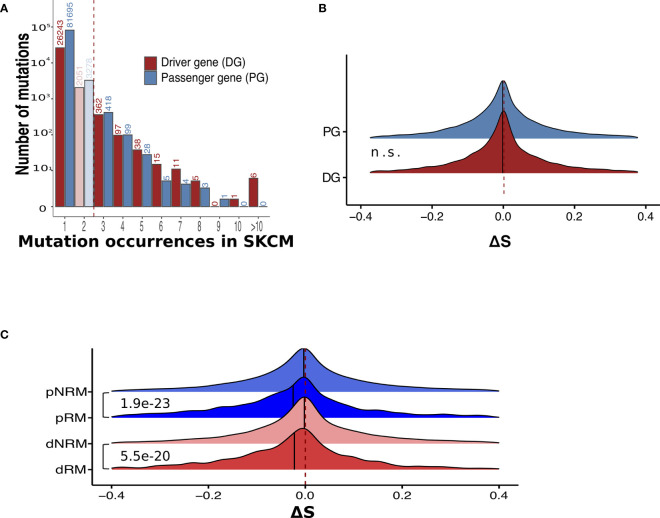
Recurrent mutations have significantly lower ΔS than non-recurrent mutations in both driver and passenger genes. **(A)** The distribution of occurrences of non-synonymous mutations in melanoma samples. Mutations presenting in driver and passenger genes are separated. **(B)** No significant ΔS difference between somatic mutations in driver and passenger genes. n.s., not significant. **(C)** Comparison of ΔS between recurrent and non-recurrent mutations in driver and passenger mutations. dRM, recurrent mutation in driver genes; dNRM, non-recurrent in driver genes; pRM, recurrent mutation in passenger genes; pNRM, non-recurrent in passenger genes. P-values were calculated using two-sided Wilcoxon rank-sum tests.

Based on the functional impact of somatic mutations, genes may be categorized into cancer driver genes and passenger genes (31). Mutations in driver genes are more likely to confer growth advantages to tumor cells and are therefore often positively selected for during cancer development (21, 32). In this study, we utilized the 1,161 melanoma driver genes (DGs) reported by Chung et al. (33) as SKCM DGs. The distributions of NS somatic mutations in DGs and passenger genes (PGs) are shown in **Figure 3A**. We detected no significant differences in MHC-I binding scores between mutations in these two gene groups (**Figure 3B** and **Figure S2A**).

By further considering the frequency of mutations in the TCGA SKCM samples, we divided the DG and PG mutations into four groups: recurrent driver mutations (dRM), non-recurrent driver mutations (dNRM), recurrent passenger mutations (pRM), and non-recurrent passenger mutations (pNRM). A comparison between these groups indicated that in both driver and passenger genes, recurrent mutations showed significantly lower ΔS than non-recurrent mutations (**Figure 3C** and **Figure S2B**). Thus, neoantigens derived from recurrent mutations tend to have lower MHC-I binding affinities with respect to peptides from their wild-type counterparts.

### The Effect of Somatic Mutations and Loss of HLA Genes

**Figure 4:**
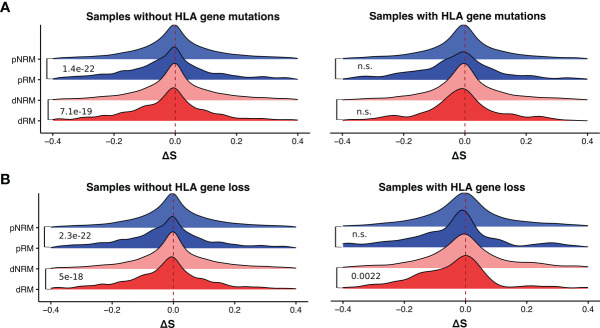
Difference in MHC-I binding affinities of somatic mutations in melanoma samples with and without HLA gene deficiency. **(A)** The distribution of ΔS of somatic mutations in samples with and without HLA gene mutations. **(B)** The distribution of ΔS of somatic mutations in samples with and without HLA gene loss. dRM, recurrent mutation in driver genes; dNRM, non-recurrent in driver genes; pRM, recurrent mutation in passenger genes; pNRM, non-recurrent in passenger genes. *P_adj_*-values were calculated using two-sided Wilcoxon rank-sum tests and adjusted for multiple testing by the Holm–Bonferroni method. n.s., not significant.

Previous studies have reported that the loss of HLA genes can affect tumor cell immune escape in lung cancer (24, 34). We thus examined the potential effect of HLA gene defects (HLA gene mutation or loss) on MHC-I binding affinity of neoantigens derived from somatic mutations in SKCM. We obtained the HLA gene mutation information of the TCGA melanoma samples from Castro et al. (11), and HLA gene loss information from Taylor et al. (35). In melanoma samples with HLA gene deficiency, the distributions of MHC-I binding affinities for dRMs, dNRMs, pRM, and pNRMs were similar to those with proficient HLA genes (**Figures 4A, B**). Significance was generally not reached when comparing the effect of HLA mutations (**Figures 4A, B**), presumably due to reduced sample size (the number of samples with HLA gene mutation or loss is small).

### Melanoma Samples Harboring Recurrent Somatic Mutations Have Lower Immune and Stromal Scores

**Figure 5:**
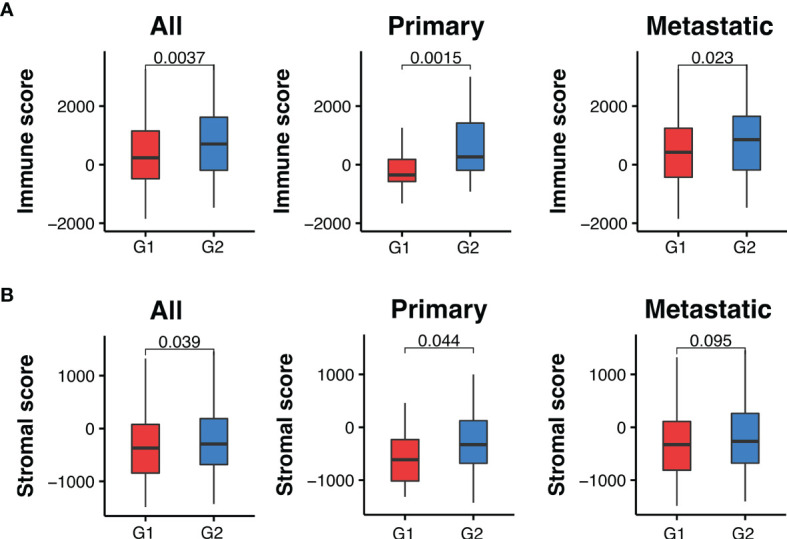
Melanoma samples with ≥7 recurrent mutations (G1) have lower immune and stromal scores than those with <7 (G2). **(A)** Comparison of immune scores between recurrent and non-recurrent mutations in all, primary and metastatic melanoma samples. **(B)** Comparison of stromal scores between recurrent and non-recurrent mutations in all, primary and metastatic melanoma samples. *P_adj_*-values were calculated using two-sided Wilcoxon rank-sum tests and adjusted for multiple testing by the Holm–Bonferroni method.

Based on the number of recurrent mutations in each sample, we divided melanoma samples into two groups. The first group contained 215 melanoma samples with at least seven recurrent somatic mutations, while the second group contained 130 samples with less than seven recurrent mutations. We compared their immune microenvironment difference by calculating sample-specific immune scores and stromal scores based on their gene expression profiles using the ESTIMATE algorithm (36). These two scores indicated the relative abundance of infiltrating immune cells and stromal cells in the tumor samples, respectively. We found that melanoma samples harboring more recurrent mutations had significantly lower immune scores compared to those with less than 7 recurrent mutations (*P_adj_* = 0.0037). This was observed in both primary and metastatic melanoma samples (*P_adj_* = 0.0015, and 0.023, respectively), as shown in **Figure 5A**. Similarly, stromal scores were also significantly lower in melanoma samples with higher levels of recurrent mutations (**Figure 5B**). These results suggest a differential tumor microenvironment between patients with high and low numbers of recurrent mutations sample groups.

### MHC-I Binding Affinities of Detected SKCM Mutations and Their Possible Alterations

In this study, the binding affinity of peptides with MHC-I complex was calculated based on the HLA genotype of the patient. For each non-synonymous somatic mutation, we also calculated MHC-I binding scores using HLA genotypes of patients that did not carry the somatic mutation (i.e., non-self HLA genotype). Comparative analyses identified a total of 46 somatic mutations that showed significantly lower ΔS with self-HLA than with non-self HLA genotypes (**Table 1**, P-value ≤0.001, two-sided Wilcoxon rank-sum test). We hypothesize that his set of mutations has been shaped by immune selection in a patient-specific manner.

**Table 1:** The mutations, which have significant lower ΔS in own patient’s HLA alleles than non-self patients’ HLA alleles, were identified in SKCM (P-value ≤0.001, two-sided Wilcoxon rank-sum test).

Gene	Mutation	ΔS	P-value
ZNF804A	R97Q	0.024951	3.85E−06
PABPC3	K177R	−0.357816	9.28E−07
CDH5	R230Q	−0.034363	1.23E−06
WHSC1	R85M	0.009242	6.74E−05
SPHK2	L302I	0.225282	1.23E−05
XRN1	L284F	−0.041268	5.46E−05
HIP1	V228I	−0.11429	2.16E−05
CSN2	P131S	−0.057814	3.08E−05
TNN	E1022K	−0.359041	4.71E−05
GIMAP7	E32K	−0.639266	4.51E−07
TMEM202	R233I	0.080141	2.48E−05
KRT34	R390Q	−0.3156	3.03E−07
ADRA2C	R409L	0.058708	4.15E−05
COL15A1	L1273F	0.122489	7.41E−08
GPRC6A	R124K	−0.231665	4.48E−05
ZNF804A	R97Q	0.037489	3.85E−06
PCDHGC3	F45L	−0.150706	8.04E−06
GALNT14	R124S	0.024075	8.84E−07
C6orf15	P152S	−0.024385	3.20E−08
MKI67	P1132S	−0.046145	4.72E−05
ZNF821	Q139K	−0.054997	2.04E−05
PCDHA13	E790K	−0.208681	4.27E−05
CSMD3	D1025N	0.079423	5.84E−09
TRIM71	D705N	0.13674	9.67E−06
HS6ST3	D246N	−0.16574	4.37E−05
RGS18	H171P	−0.53604	2.56E−06
GIMAP7	E32K	0.332249	4.51E−07
HIF3A	P646S	0.044866	3.22E−05
SGMS1	I87N	−0.15749	3.28E−05
FBXO24	R377Q	−0.002459	6.11E−05
LRP1B	D1612N	0.056829	2.75E−06
F2RL3	E346K	−0.02666	1.24E−07
QRSL1	V294I	0.004044	1.38E−08
SAA1	A99T	0.021469	4.36E−05
PXDN	V808I	−0.217944	1.57E−09
SPIDR	E399G	−0.514393	5.90E−11
CATSPERB	M414I	−0.092106	2.18E−05
PPAPDC2	A262V	−0.182649	3.98E−06
LCT	P1458S	0.393053	2.20E−05
REEP4	P170S	−0.065841	1.31E−05
ZNF592	P564L	0.210062	5.02E−05
MLLT6	S594C	−0.291905	1.14E−05
MRPL37	P288L	−0.092613	2.29E−05
PCLO	R3857K	−0.031115	1.72E−07
PTPN4	R838Q	−0.127347	2.53E−05
MYBPC2	S602N	−0.235127	2.43E−07

**Figure 6:**
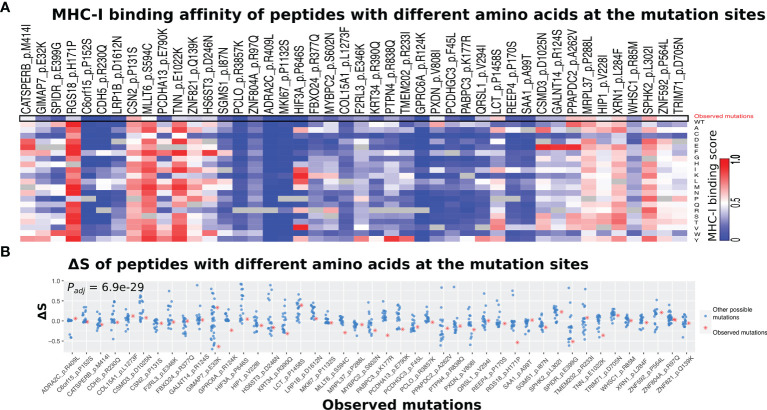
Comparison of MHC-I binding alterations between observed and other possible amino acid changes. **(A)** Heatmap showing a list of 46 somatic mutations that derive neoantigen with significantly lower ΔS using the self HLA genotype than non-self HLA genotypes. The HLA genotypes of melanoma patients that do not have a specific mutation were selected as non-self. **(B)** The observed amino acid changes (red dots) tend to have lower ΔS than those unobserved ones (other 18 possible amino acid changes) at the same position. The *P_adj_*-value was calculated by the ANOVA test and adjusted for multiple testing by the Benjamini–Hochberg method.

Next, we examined whether the observed amino acid change for a given somatic mutation tended to have a different MHC-I binding affinity compared with the other 18 possible amino acid changes. As shown in **Figure 6A**, our result indicated that the observed amino acid change tended to be lower binding affinity than other possible changes. This trend is more obvious when comparing ΔS for the observed and other amino acid changes (**Figure 6B**). Indeed, the ΔS values for the observed changes were significantly lower than those for the other possible amino acid changes (*P_adj_* = 6.9e−29, ANOVA test and adjusted for multiple testing by the Benjamini–Hochberg method).

## Discussion

In this study, we demonstrated that T cell-mediated immunosurveillance likely contributes to the mutational landscape in SKCM tumors. Here we used the MHC-I binding difference between mutation-derived neoantigens (*S_mu_*) and their corresponding wild-type peptides (*S_wt_*) to measure the effect of mutations on antigenicity, i.e., ΔS = *S_mu_* − *S_wt_*. This value has been defined as differential aggretope index (DAI) in previous analysis by Duan et al. (8). Following that, DAI has been used in several studies (8, 9, 37–40) to measure the antigenicity of neoantigens, using the same formula or a variant version (e.g., *S_mu_*/*S_wt_*) (39). DAI has been used to select the best potential immunoprotective neoepitopes (i.e., those with the highest DAI values) from a vast number of somatic mutations (8). Sedlacek et al. reported that in the absence of CD91 on dendritic cells there was a rise of neoepitopes with high DAI, suggesting the function CD91 during immunosurveillance (40). In this study, by showing the ΔS difference between recurrent and non-recurrent somatic mutation derived neoantigens, we showed the potential effect of immunosurveillance on shaping the somatic mutation landscape. In addition, we have used another DAI metric (*S_mu_*/*S_wt_*) to compare recurrent and non-recurrent mutations, and observed consistent results as ΔS (**Figure S3**).

In this study, we defined recurrent somatic mutations as those occurring in at least three melanoma patients. To evaluate the robustness of our analysis, we have also applied different thresholds (the number of patients with a specific somatic mutation) to define recurrent mutations. Specifically, we defined recurrent mutations as those presenting in ≥s (s = 3, 4, 5, 6, 7) different melanoma patients, and compared with non-recurrent mutations. We observed significant lower ΔS for recurrent mutations regardless of threshold setting (**Figure S4**).

**Figure 7:**
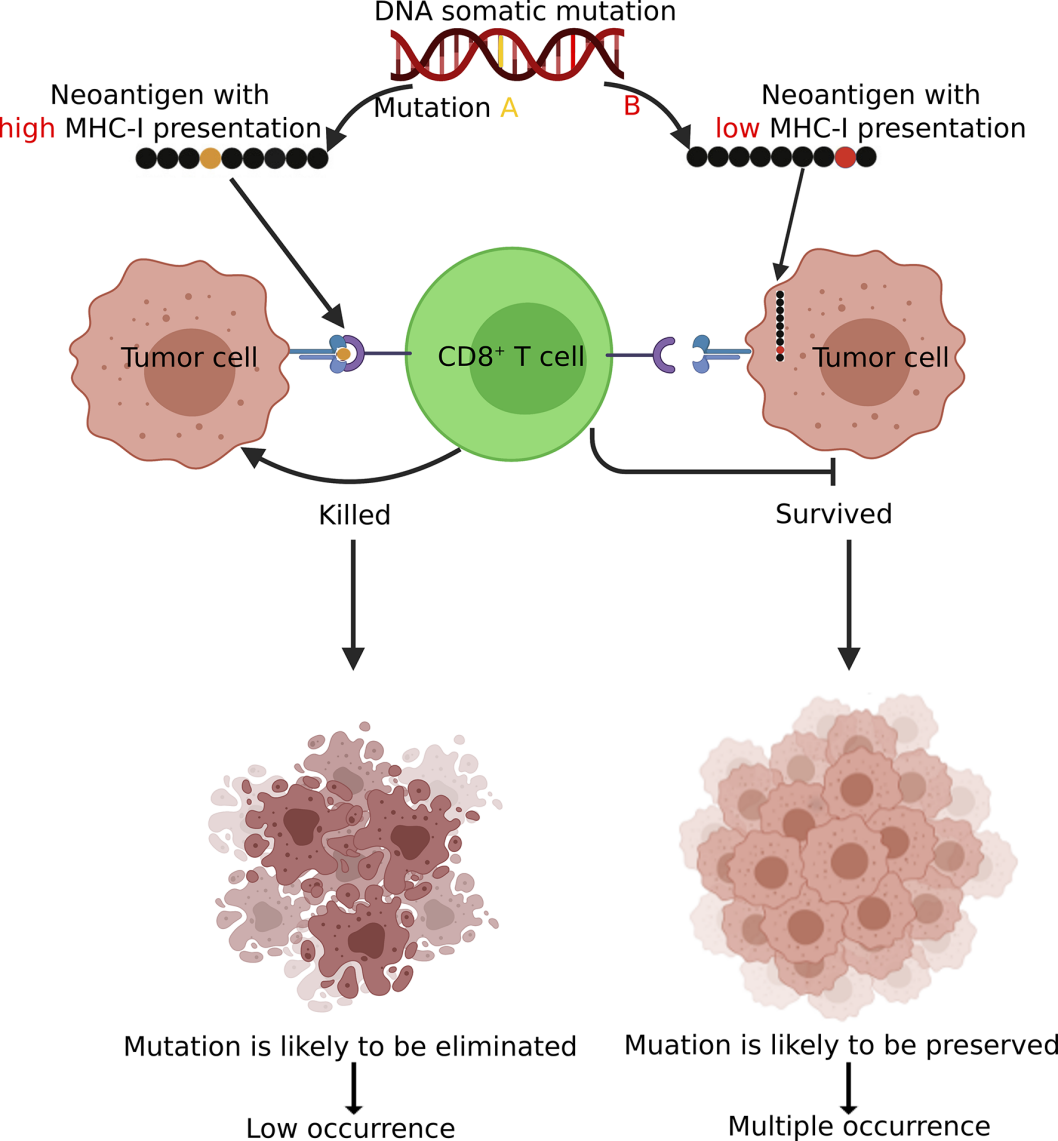
A schematic diagram demonstrating how immunosurveillance shapes the somatic mutation landscape in tumors. Somatic mutations leading to CD8+ neoantigen with high MHC-I binding affinity are more likely to be recognized and eliminated by T cells and there tend to have low occurrences (non-recurrent) in tumor. In contrast, mutations with low MHC-I binding affinity are more likely to be preserved and present in multiple patients. Figure created using BioRender (https://biorender.com/).

Our analysis indicated that neoantigens derived from recurrent mutations tended to have lower binding affinity with the MHC-I complex compared to those from non-recurrent mutations. We found that in both driver and passenger genes, recurrent mutations tended to have reduced MHC-I binding affinities compared to non-recurrent mutations (**Figure 3** and **Figure S2**). These results support the model (**Figure 7**) in which tumor cells presenting neoantigens with strong MHC-I binding capacity are more likely to be eliminated during tumorigenesis and are less likely to be observed (i.e., non-recurrent) at the population level. In contrast, somatic mutations that result in neoantigens with low MHC-I binding affinity are under less selective pressure by the host immune system and are therefore more likely to be observed in tumors. This model is consistent with observations from previous studies (41–45).

## Conclusion

Neoantigens derived from recurrent mutations generally have low binding affinities with MHC-I complex. Based on this observation and previous studies, we proposed a model to explain how T cell-mediated immunosurveillance shapes the mutational landscape in tumors.

## Materials and Methods

### Data Preparation and Processing

MuTect2-called whole-exome sequencing (WES) mutation annotation format (MAF) files of the SKCM from The Cancer Genome Atlas (TCGA) were downloaded from the Genomic Data Commons (GDC) data portal (https://portal.gdc.cancer.gov; data release v7). From these files, we selected all non-synonymous mutations for downstream analysis.

The TCGA level 3 gene expression data of melanoma were obtained from the Genomic Data Commons (GDC, available at: https://portal.gdc.cancer.gov/) Data Portal on Mar 7, 2019. From the GDC, we downloaded the expression profiles for tumors with the disease type “melanoma” from the “TCGA-SKCM”. Fragments per kilobase of exon per million reads mapped (FPKM) were used for expression quantification for a total of 20,501 protein-coding genes annotated in the TCGA data portal.

### MHC-I Binding Affinity Predictions

HLA affinities of mutated and matched-wild-type peptides were predicted for their sample-specific HLA genotype (a specific combination of two HLA-A, two HLA-B, and two HLA-C alleles) using NetMHCpan4.0 (25). Firstly, for each type of HLA allele (HLA-A, HLA-B, and HLA-C), many subtypes exist and most of these HLA allele subtypes account for a very small proportion of the population (≤0.01%). To ensure stability and avoid over-calculation, all HLA allele subtypes with a proportion greater than 0.01% were selected in this study. A total of 92 HLA alleles were selected for the peptide binding HLA allele prediction. Thirty subtypes of HLA-A alleles cover 99.76% of the population; 39 subtypes of HLA-B alleles cover 99.08% of the population; 23 subtypes of HLA-C alleles cover 99.81% of the population. The derived frequencies for each HLA allele were compared with the allele frequencies from a healthy US blood donor population, downloaded from the Allele frequency net (46), **Supplementary Table 3**). Second, each mutated and matched wild-type peptide was predicted for the 92 HLA alleles. Finally, the patient-specific MHC-I binding affinities of each mutated and matched wild-type peptides were obtained.

### Calculation of Patient-Specific MHC-I Binding Affinity of Somatic Mutations

We used an MHC-I binding score to represent the binding affinity of a non-synonymous mutation with patient-specific HLA class I alleles (two HLA-A, two HLA-B, and two HLA-C) (**Figure 1**). First, for each non-synonymous mutation, we enumerated the nine 9-mers (peptides with 9 amino acids) that overlap with the mutated residue and applied NetMHCPan4.0 (25) to calculate their binding affinity with the MHC-I complex using patient-specific HLA class I alleles (two HLA-A, two HLA-B, and two HLA-C). Second, we calculated the maximum value for each HLA allele to capture the peptide with the highest binding affinity, resulting in six HLA allele-specific affinity scores for each mutation. Finally, we selected the maximum value to represent the best presentation potential by the MHC-I complex for peptides derived from a specific non-synonymous mutation. The HLA typing of all the TCGA-SKCM samples was downloaded from the TCIA (ref link: https://tcia.at/home).

To measure the effect of somatic mutations on antigenicity, the MHC-I binding difference between mutation-derived neoantigens (*S_mu_*) and their corresponding wild-type peptides (*S_wt_*) was calculated, denoted by ΔS = *S_mu_* − *S_wt_*. This definition is consistent with the differential aggretope index (DAI) introduced in the previous studies by Duan et al. (8, 9, 40).

### Evaluation of the MHC-I Binding Prediction Pipeline

To test the performance of affinity scores representing actual MHC class I presentation, we downloaded independent mass spectrum data of 5 different human cell lines (Fibroblast, SupB15W, JY, HCC1937, and HCC1143) from Bassani-Sternberg et al. (27). These peptides were observed in complex with MHC-I on the cell surface across known HLA alleles. An independent non-MHC-binding peptide dataset was downloaded from Abelin et al.; these peptides were validated to not bind with known MHC-I alleles in mass spectrometry experiments (26). Detailed information on the classification of MHC-binding peptides and independent non-MHC-binding peptide controls are shown in **Supplementary Tables 1A–G**.

### Estimation of Stromal and Immune Scores

Sample-specific immune scores and stromal scores were calculated based on gene expression profiles (described above). The ESTIMATE algorithm (36) was applied to the normalized expression matrix for estimating the stromal and immune scores for each melanoma sample.

### Statistical Analysis

The R statistical package was used for all data processing and statistical analysis (R package: stats v3.6.2). All details of the statistical tests are specified in the associated text or figure legends. For the comparison of the observed mutations and their according other possible mutations, the P-value was calculated by using an ANOVA test and adjusted for multiple testing by the Benjamini-Hochberg method. For the other statistical analyses, P-values were calculated by using the “Wilcox_test” function from the R package: stats v3.6.2, which applies the two-sided Wilcoxon rank-sum test and corrected multiple testing using the Holm–Bonferroni method. A statistically significant difference was assumed when adjusted P ≤0.05.

## Data Availability Statement

The original contributions presented in the study are included in the article/**Supplementary Material**. Further inquiries can be directed to the corresponding author.

## Author Contributions

CC conceived the project. CJ, HW, and CC performed computational analyses. CJ, CC, ES, and KZ wrote the manuscript. CJ, C-CC, ES, and CC interpreted the results. CC supervised the project. All authors contributed to the article and approved the submitted version.

## Funding

This work is supported by the Cancer Prevention Research Institute of Texas (CPRIT; RR180061, to CC) and the NCI of the NIH (1R21CA227996, to CC), and the T32 training grant of the NIH (T32 AI007363, to ES). CC is a CPRIT Scholar in Cancer Research.

## Conflict of Interest

Author C-CC was employed by the company Chempartner Corporation.

The remaining authors declare that the research was conducted in the absence of any commercial or financial relationships that could be construed as a potential conflict of interest.

## Publisher’s Note

All claims expressed in this article are solely those of the authors and do not necessarily represent those of their affiliated organizations, or those of the publisher, the editors and the reviewers. Any product that may be evaluated in this article, or claim that may be made by its manufacturer, is not guaranteed or endorsed by the publisher.

## Abbreviations

MHC, major histocompatibility complex; MHC-I, MHC class I; HLA, human leukocyte antigen; dRM, recurrent mutation in driver genes; dNRM, non-recurrent mutation in driver genes, pRM, recurrent mutation in passenger genes; pNRM, non-recurrent mutation in passenger genes; WES, whole-exome sequencing); GDC, Genomic Data Commons; ROC, Receiver Operating Characteristics; AUC, Area Under The Curve.
