# Supplementary material for: Influence of T Cell-Mediated Immune Surveillance on Somatic Mutation Occurrences in Melanoma
Source: Front Immunol. 2022 Jan 17;12:703821. doi: 10.3389/fimmu.2021.703821 (PMC8801458; doi:10.3389/fimmu.2021.703821)
Supplement: Supplementary file 1 [file DataSheet_1.zip › Supplemental Materials/frontiers_SupplementaryMaterial_Jiang_et_al.pdf]

Supplementary Material

1 FIGURE S1.

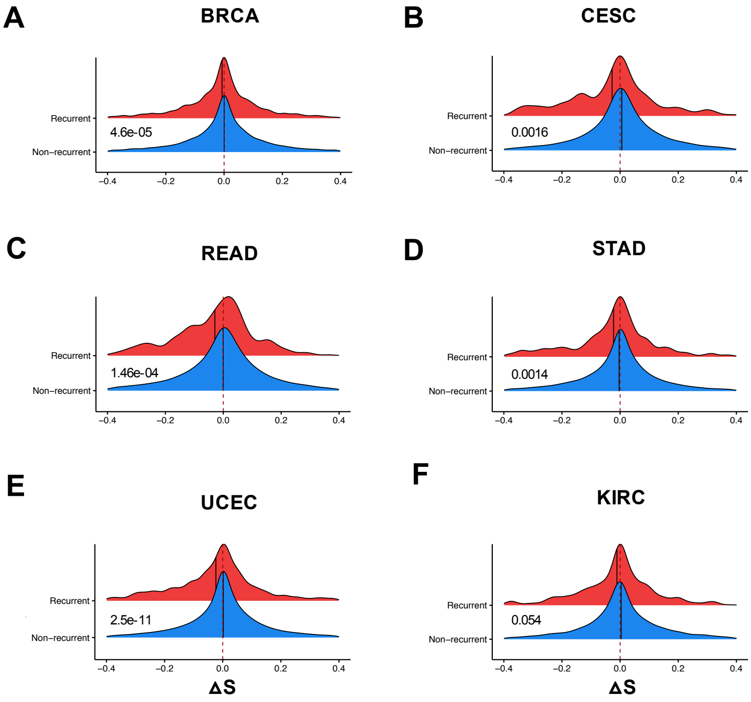

Figure S1. Neoantigens derived from recurrent mutations tend to have lower binding affinity than those from non-recurrent mutations A. BRCA. B. CESC. C. READ. D.STAD. E.UCEC. F. KIRC. The P_{adj} values were calculated by using two-sided Wilcoxon rank-sum test and adjusted for multiple testing by the Holm–Bonferroni method.

2 FIGURE S2.

We used the cancer driver genes from another public database [1]. We observed consistent results as the Fig.3.

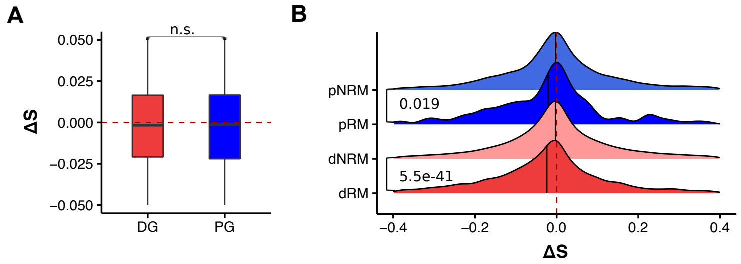

Figure S2. A different set of driver genes [1] was used for validating the results of Figure.3. A. Recurrent vs non-recurrent mutations in SKCM. B. Compare the 4 groups difference by max.score of ΔS. The P_{adj} values were calculated by using two-sided Wilcoxon rank-sum test and adjusted for multiple testing by the Holm–Bonferroni method.

3 FIGURE S3.

we re-examined the analysis using the DAI metric proposed in Rech et al (DAI = S_{mu} / S_{wt}), which result in the same conclusion as with ΔS.

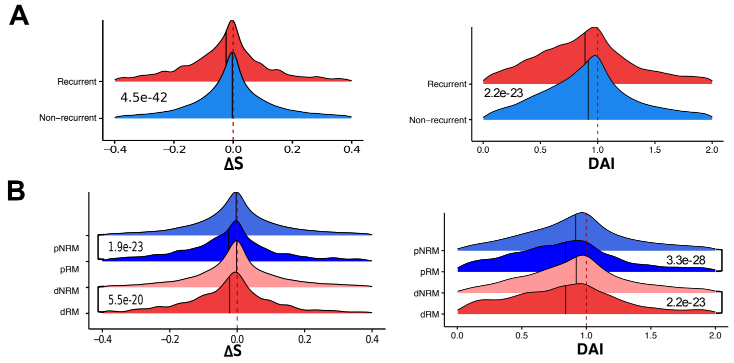

Figure S3. the DAI metric proposed in Rech et al [2] (DAI = S_{mu} / S_{wt}), which result in the same conclusion as with ΔS. A. Left ridgeline plot showing the ΔS difference between the recurrent mutations and non-recurrent mutations. Righ ridgeline plot showing the DAI difference between the recurrent mutations and non-recurrent mutations. B. Left ridgeline plot showing the ΔS difference in the four groups (dRM, dNRM, pRM, and pNRM). Righ ridgeline plot showing the DAI difference in the four groups (dRM, dNRM, pRM, and pNRM). The P_{adj} values were calculated by using two-sided Wilcoxon rank-sum test and adjusted for multiple testing by the Holm–Bonferroni method.

4 FIGURE S4.

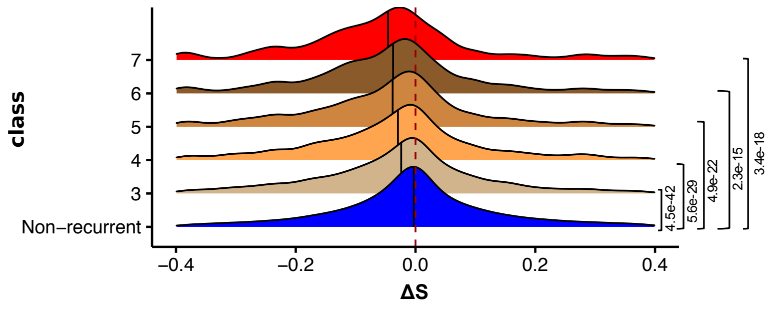

Figure S4. The ΔS of recurrent mutations are significant lower than the non-recurrent mutations regardless of threshold setting. The recurrent mutations were defined as those presenting in $\geq$ s (s=3, 4, 5, 6, 7) different melanoma patients. The P_{adj} values were calculated by using two-sided Wilcoxon rank-sum test and adjusted for multiple testing by the Holm–Bonferroni method.

REFERENCES

1. integrative Onco Genomics. https://www.intogen.org/search

2. Rech, A. J., Balli, D., Mantero, A., Ishwaran, H., Nathanson, K. L., Stanger, B. Z., Vonderheide, R. H. (2018). Tumor Immunity and Survival as a Function of Alternative Neopeptides in Human Cancer. Cancer Immunology Research, 6(3), 276–287.
