# Supplementary figures and images for: Influence of T Cell-Mediated Immune Surveillance on Somatic Mutation Occurrences in Melanoma
Source: Front Immunol. 2022 Jan 17;12:703821. doi: 10.3389/fimmu.2021.703821 (PMC8801458; doi:10.3389/fimmu.2021.703821)

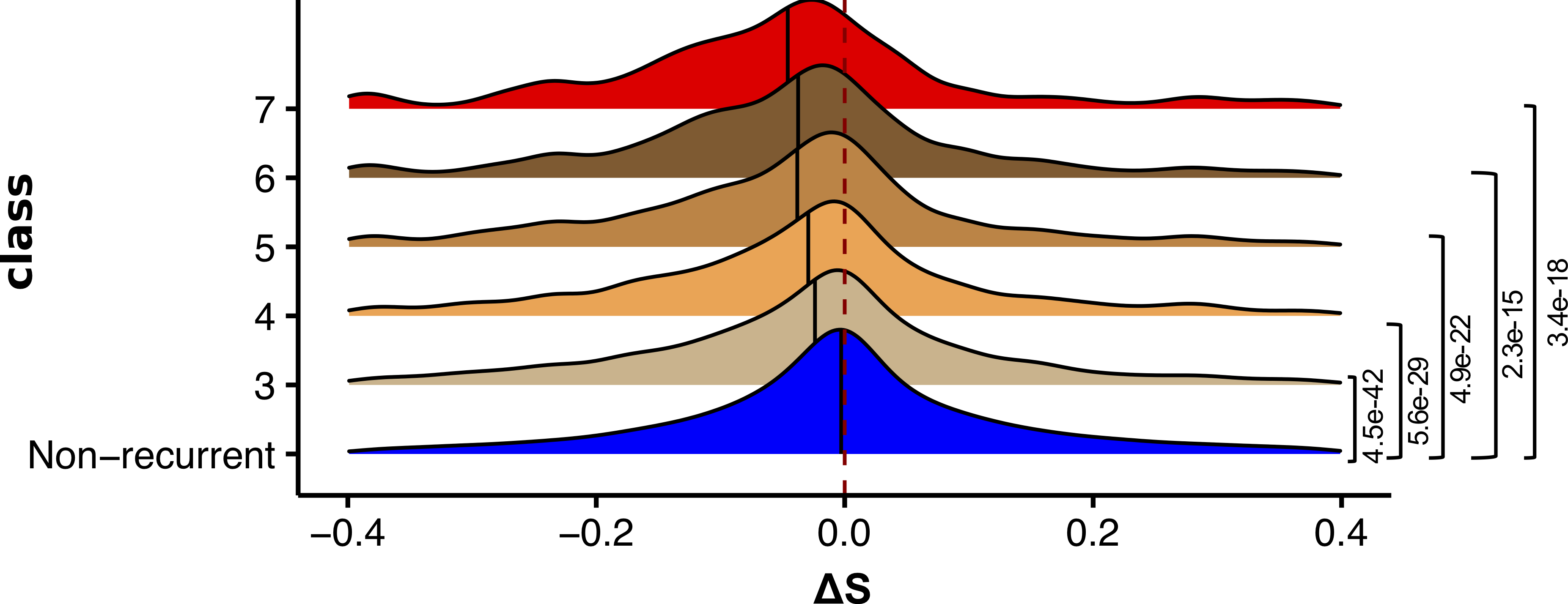

Supplement: Supplementary file 1 [file DataSheet_1.zip › Supplemental Materials/Figures/FigS4.tiff]

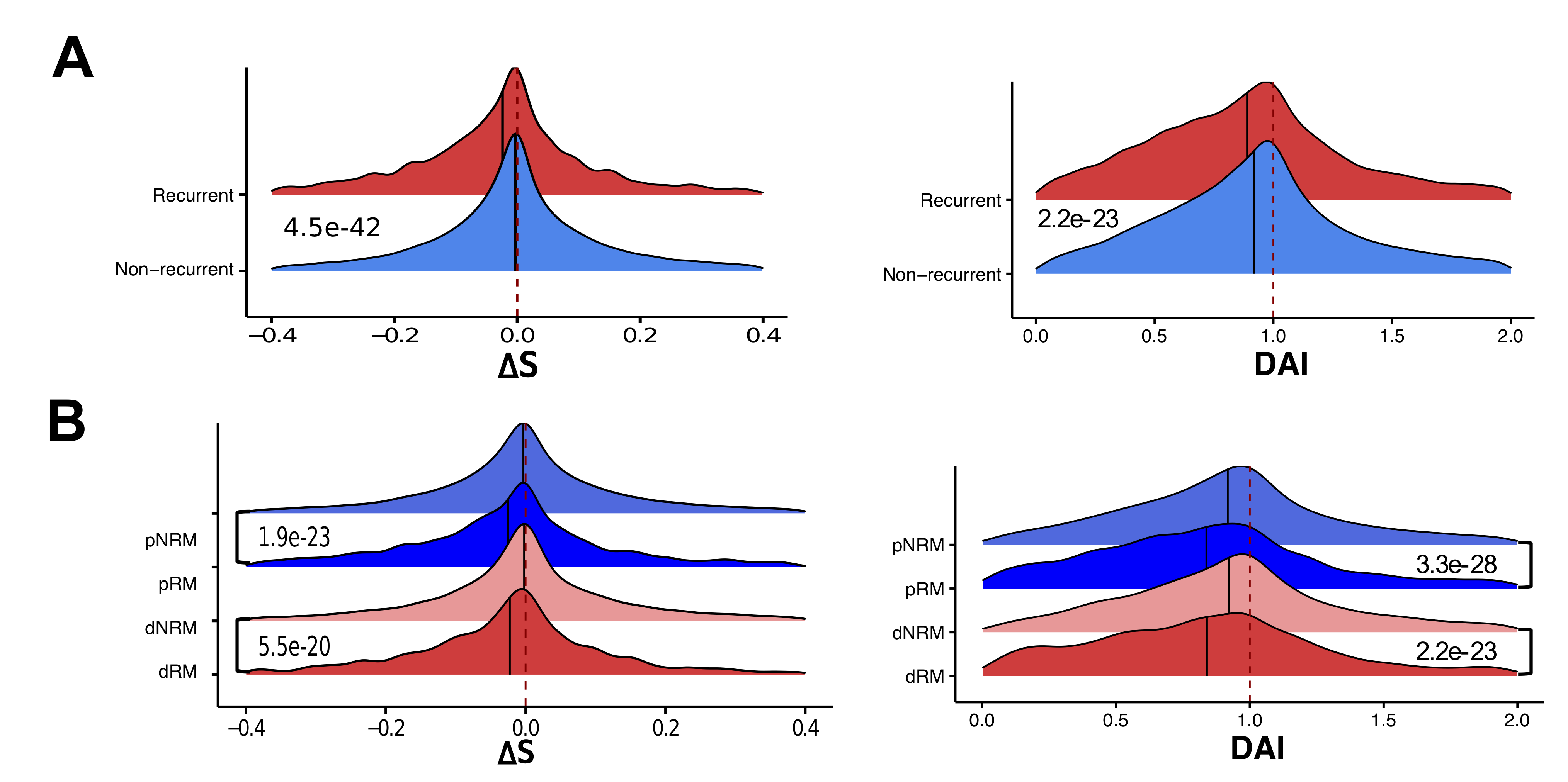

Supplement: Supplementary file 1 [file DataSheet_1.zip › Supplemental Materials/Figures/FigS3.tiff]

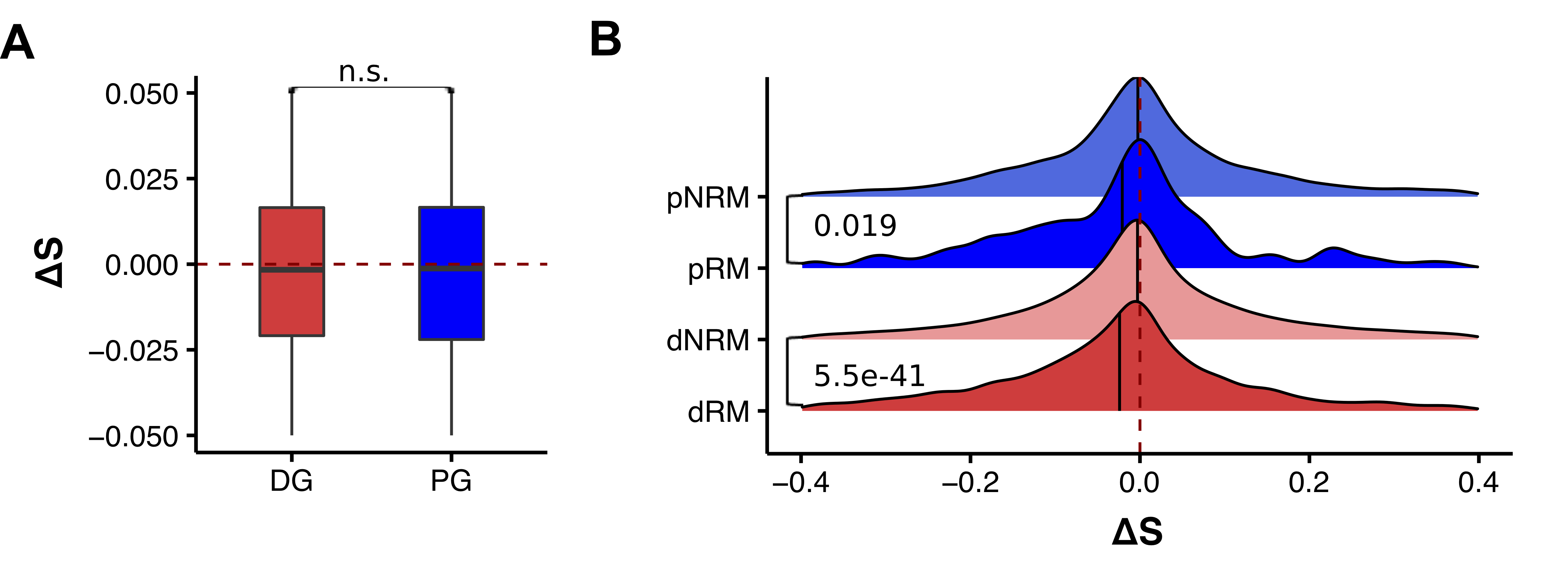

Supplement: Supplementary file 1 [file DataSheet_1.zip › Supplemental Materials/Figures/FigS2.tiff]

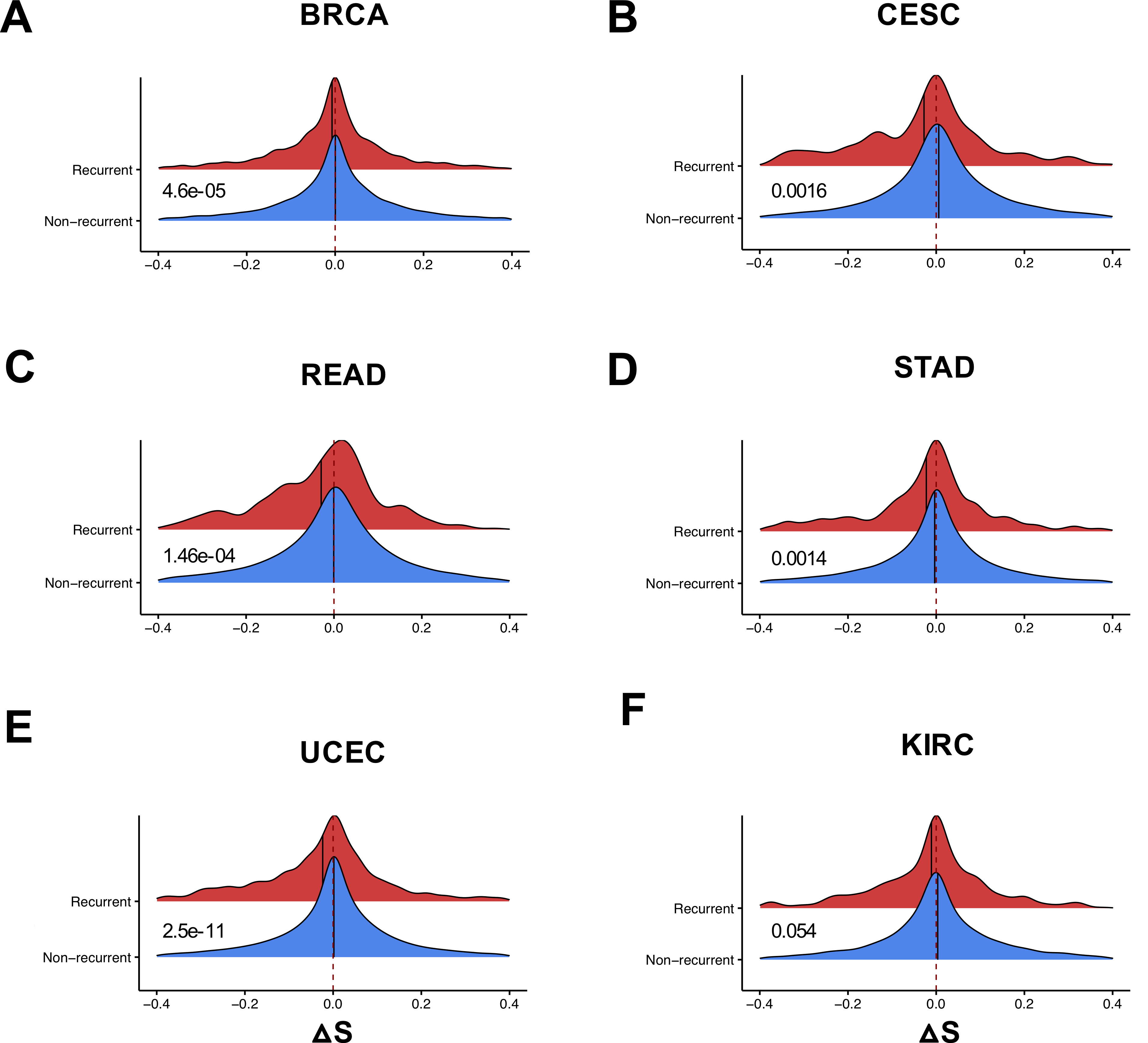

Supplement: Supplementary file 1 [file DataSheet_1.zip › Supplemental Materials/Figures/FigS1.tiff]
